# Systemic Catheter-Related Venous Thromboembolism in Children: Data From the Italian Registry of Pediatric Thrombosis

**DOI:** 10.3389/fped.2022.843643

**Published:** 2022-03-23

**Authors:** Donatella Lasagni, Margherita Nosadini, Angelo Claudio Molinari, Paola Saracco, Maria Federica Pelizza, Fiammetta Piersigilli, Maria Caterina Putti, Marcella Gaffuri, Paola Giordano, Giulia Lorenzoni, Andrea Francavilla, Sandra Trapani, Matteo Luciani, Agnese Suppiej, Antonella Tufano, Daniela Tormene, Matteo Martinato, Dario Gregori, Stefano Sartori, Paolo Simioni, The Systemic Thromboses Working Group of the Italian Registry of Pediatric Thrombosis (RITI Registro Italiano Trombosi Infantili), Manuela Agostini

**Affiliations:** ^1^Pediatric Unit, Meyer Children's University Hospital, Florence, Italy; ^2^Paediatric Neurology and Neurophysiology Unit, Department of Women's and Children's Health, University Hospital of Padua, Padua, Italy; ^3^Thrombosis and Hemostasis Unit, G. Gaslini Children's Hospital, Genoa, Italy; ^4^Pediatric Hematology, Department of Pediatrics, University Hospital Città della Salute e della Scienza, Turin, Italy; ^5^Neonatology Unit, IRCCS Pediatric Hospital Bambin Gesù, Rome, Italy; ^6^Department of Oncology and Hematology, University Hospital of Padua, Padua, Italy; ^7^Pediatric Department Civile Maggiore Hospital, Verona, Italy; ^8^Pediatric Hematology and Oncology, University of Bari, Bari, Italy; ^9^Unit of Biostatistics, Epidemiology and Public Health, Department of Cardiac, Thoracic, Vascular Sciences, and Public Health, University of Padova, Padua, Italy; ^10^Department of Health Sciences, Meyer Children's University Hospital, Florence, Italy; ^11^Department of Hematology and Oncology and Transfusional Medicine, IRCCS Pediatric Hospital Bambin Gesù, Rome, Italy; ^12^Department of Medical Sciences-Pediatric Section, University of Ferrara, Ferrara, Italy; ^13^Department of Clinical Medicine and Surgery, University of Naples, Naples, Italy; ^14^General Internal Medicine and Thrombotic and Hemorrhagic Diseases Unit, University of Padua Medical School, Padua, Italy

**Keywords:** thrombosis, catheter-complications, central venous catheter (CVC), pediatric, children, registry, heparin

## Abstract

**Background:**

Central venous catheters (CVCs) represent one of the main risk factors for venous thrombotic events (VTEs) in children.

**Methods:**

We studied the Italian Registry of Pediatric Thrombosis (RITI) with regard to systemic radiologically confirmed CVC-related VTEs (CVC-VTEs) occurred during 6.5 years in children aged 29 days to 18 years.

**Results:**

A total of 78 CVC-VTEs were included, which occurred in 76 patients (40/76, 53% males). CVC-VTEs comprised 67 non-cardiac VTEs (86%) and 11 intracardiac thrombotic events (ICTEs) (14%); the median age at onset was 19 and 17 months, respectively. The most frequent reason for CVC insertion was supportive therapy. The catheters were placed percutaneously in 85% of cases (56/66) and surgically in the remaining 15% (10/66). Peripherally inserted central catheters (PICCs) were used in 47% (31/66) cases, partially implanted catheters in 42% (28/66), non-implantable catheters in 7% (5/66), and totally implanted catheters (Port) in 2% (1/66). CVC-VTEs were symptomatic in 77% of cases (60/78), while in the remaining 23%, they were incidentally detected on the imaging performed for the underlying condition. The median time between CVC insertion and the onset of symptoms was 10 days in non-cardiac VTEs and 39 days in ICTEs. Doppler ultrasound was the diagnostic technique most frequently used. The venous compartment most frequently affected was the veins of the lower extremities (52%, 43/73). Anti-thrombotic treatment was administered in 96% of CVC-VTEs (75/78). About 2.6% (2/76) of patients experienced a second thrombotic event. At discharge, post-thrombotic syndrome was reported in 13.5% (5/37) events with available data, CVC replacement in 10.8% (4/47), and ischemic necrosis with toe finger amputation in 2.7% (1/37). Three patients died due to an underlying condition; no CVC-VTE-related deaths were reported.

**Conclusions:**

We have carried out a registry-based study on CVC-VTEs in the children in Italy, providing the data that may help improve the detection and management of this CVC-related complication.

## Introduction

Central venous catheters (CVCs) have greatly improved the care of patients requiring long-term venous access (1) and are widely used in children with severe conditions such as cancer, sepsis, prematurity, and chronic diseases, to deliver intravenous fluids, total parenteral nutrition, and therapies (2). The use of CVCs may be burdened by severe complications, first of all, systemic CVC-related venous thrombotic events (CVC-VTEs) (3). In particular, the presence of a CVC is the single most common risk factor for VTE in children, often accompanied by other concomitant risk factors (1, 4–6). CVC-VTE is defined as a mural thrombus extending from the catheter into the lumen of a vessel and leading to partial or total catheter occlusion, with or without clinical symptoms (2, 7). CVC-VTEs may be symptomatic, with signs of inflammation or vascular obstruction (such as swelling, pain, and discoloration), or completely asymptomatic, detected only by imaging techniques; a CVC dysfunction (inability to aspirate and to infuse) may be the only sign of CVC-VTE (2, 8–10). CVC-VTEs, especially when they involve lower extremities, can lead to acute life-threatening complications such as pulmonary embolism (PE), or long-term complications like post-thrombotic syndrome (PTS), a syndrome of chronic venous insufficiency following deep venous thrombosis; signs and symptoms include pain, vein dilation, edema, skin pigmentation, and venous ulcers (2, 8).

Although the relevant morbidity and mortality in CVC-VTE are well recognized (11), and a higher number of VTEs are related to CVC in the pediatric population than in adults (11, 12), limited data are available in children. The available pediatric guidelines for the prophylaxis, diagnosis, and treatment of systemic CVC-VTE (6) are largely extrapolated from the studies on adults (13), although age-related differences in coagulation and cardiovascular status exist (14).

To improve the knowledge concerning the risk factors and management of systemic CVC-VTE in Italian children, we analyzed pertinent dataon pediatric CVC-VTEs collected in the Italian Registry of Pediatric Thrombosis (RITI, Registro Italiano Trombosi Infantili).

## Materials and Methods

The RITI is an event-based nationwide registry including cerebral and systemic thrombotic events in neonates and children (http://www.trombosinfantili.it/). After the written consent of the parents was obtained, events are enrolled in the RITI by the Italian physicians who voluntarily contribute to the registry. The RITI has been approved by the Ethical Committee of University Hospital of Padua, Italy (1653P). Further details on the RITI structure are available in previously published RITI reports on pediatric cerebral thrombosis (15) and neonatal and pediatric systemic thrombosis (10, 16).

The data included in the present report are relative to pediatric (age 29 days−18 years) systemic radiologically confirmed CVC-VTEs enrolled in the RITI, which occurred during 6.5 years (between January 1, 2007 and October 30, 2013), and include non-cardiac VTEs and intracardiac thrombotic events (ICTEs). The data were retrospectively collected and analyzed. Continuous variables were reported as median and interquartile range (IQR). Due to data availability, the denominators in the Results section may vary.

## Results

### Demographics and Clinical Data

During the study period, 78 systemic CVC-VTEs were enrolled in the RITI: 67 non-cardiac VTEs (86%, 67/78) and 11 ICTEs (14%, 11/78). The 78 CVC-VTEs occurred in 76 patients (40/76, 53%: males; 74/76, 97%: Caucasian); of these, 2 experienced a second thrombotic event. The median age at onset was 19 months (IQR 75) for non-cardiac VTEs and 17 months (IQR 94) for ICTEs.

The most frequent underlying conditions or risk factors included malignancies (19/78, 24%), infections (15/78, 19%), and cardiac disease (8/78, 10%) (Table 1).

**Table 1:** General data on admission ward where the thrombosis occurred, underlying disease and risk factors, and reason for CVC insertion. Data on associated risk factors were available in 85% (66/78).

**Admission ward where the thrombosis occurred**	***n* (%)**
PICU	24/78 (31%)
Oncology unit	19/78 (24%)
Pediatric general ward	16/78 (21%)
Pediatric specialist ward (cardiology, nephrology, infectious disease)	12/78 (15%)
Surgical ward	7/78 (9%)
**Underlying diseases/risk factors**	***n*** **(%)**
Malignancies	19/78 (24%)
Infections	15/78 (19%)
Heart disease	8/78 (10%)
Renal failure	6/78 (8%)
Malformative disease	6/78 (8%)
Neurometabolic disease	6/78 (8%)
Dehydration	5/78 (6%)
CVC only	13/78 (17%)
**Reason for CVC insertion (multiple for each event)**	
Supportive therapy	30/78 (38%)
Total parenteral nutrition	27/78 (35%)
Blood samples	27/78 (35%)
Chemotherapy	18/78 (23%)
Surgical procedures	7/78 (9%)
Hemodialysis	9/78 (12%)

*CVC, central venous catheter; PICU, pediatric intensive care unit*.

Supportive therapy was the most frequent reason for CVC insertion overall (Table 1). For ICTEs, chemotherapy was the most frequent reason for CVC insertion (8/11, 73%), followed by supportive therapy (3/11, 27%).

CVC-VTEs were symptomatic in 77% of cases (60/78); in 6 of these (4 non-cardiac VTEs, 2 ICTEs), CVC malfunctioning was the only clinical sign. CVC-VTEs were completely asymptomatic in the remaining 23% of cases (18/78: 9 non-cardiac VTEs, 9 ICTEs) and were incidentally detected on the imaging performed for underlying diseases (mostly oncological and surgical) (Table 2). None of the patients developed PE.

**Table 2:** Symptoms of CVC-VTE and diagnostic investigations.

	**Non-cardiac VTE (*n* = 67)**	**ICTE (*n* = 11)**
**Signs and symptoms**°
Edema	43/67 (64%)	
Pain	18/67 (27%)	
Cyanosis/dyschromia	18/67 (27%)	
Superficial collateral vein	5/67 (7%)	
Cava inferior syndrome	4/67 (6%)	
Chilotorax	3/67 (4%)	
CVC malfunctioning	4/67 (6%)	2/11 (18%)
Diagnostic imaging^*^	**Non-cardiac VTE**	**ICTE**
**Symptomatic**	**(*****n*** **=** **58)**	**(*****n*** **=** **2)**
D-US	48/58 (83%)	
CT-angiography	6/58 (10%)	
MR-angiography	2/58 (3%)	
Venography	2/58 (3%)	
Echocardiography		2/2 (100%)
**Asymptomatic**	**(*****n*** **=** **9)**	**(*****n*** **=** **9)**
D-US	8/9 (89%)	
CT-angiography	1/9 (11%)	
MR-angiography	1/9 (11%)	
Echocardiography	2/9 (22%)	9/9 (100%)

*CVC-VTEs were symptomatic in 77% of cases (60/78) and asymptomatic in 23% (18/78: 9 non-cardiac VTEs, 9 ICTEs).Data on clinical presentation were available in 79% (62/78). CT, computed tomography; D-US, Doppler ultrasound; MR, magnetic resonance imaging. ° >1 symptom for each patient*.

* *In 11 cases, multiple diagnostic techniques were performed to confirm diagnosis. Venography was performed in 2 patients with severe venous lower limb thrombosis*.

### CVC-VTE Diagnosis and Location

To document a suspected thrombotic event in the 60 symptomatic CVC-VTEs, the diagnostic investigations carried out included Doppler ultrasound (D-US), echocardiography, computed tomography (CT) and magnetic resonance imaging (MRI) with contrast agent, and venography (Table 2). D-US was considered positive for CVC-VTEs according to the standardized criteria: intraluminal material, dilatation and/or non-compressibility of the vessel, and an abnormal color Doppler pattern in the vessel (17, 18).

The median time between CVC insertion and the onset of symptoms was 10 days (IQR 23) in non-cardiac VTEs and 39 days (IQR 34) in ICTEs.

The clot location was available in 94% of cases (73/78) (Table 3A): the venous compartment most frequently affected was the veins of the lower extremity (38/73, 52%), particularly the femoral veins (21/73). All CVC-VTEs were unilateral (right side in 53/78, left side in 25/78) on the CVC side.

**Table 3A:** CVC location and thrombosis site.

**CVC location**		***n* (%)**	**Clot location**
**Lower vascular system**	Femoral vein	38/73 (52%)	Femoral vein 21 Iliac-femoral vein 12 Femoral-iliac-inferior cava vein 3 Tibial vein 1 Iliac vein 1
**Upper vascular system**	Basilica vein	2/73 (3%)	Jugular vein 1 Brachial vein 1
	Cephalic vein	2/73 (3%)	Jugular-subclavian 1 Brachial vein 1
	Brachial vein	3/73 (4%)	Brachial vein 2 Superior cava vein 1
	Subclavian vein	9/73 (12%)	Right atrium 4 Jugular-subclavian 2 Subclavian vein 1 Jugular vein 1 Superior cava vein 1
	Jugular vein	19/73 (26%)	Jugular vein 9 Right atrium 6 Jugular-subclavian 2 Jugular-superior cava vein 2

*CVC location and site of thrombosis (data available in 73/78)*.

### CVC Characteristics

Available CVC data are summarized in Table 3B. CVCs were placed percutaneously in 85% of cases (56/66) and surgically in the remaining 15% (10/66). The line was a peripherally inserted central catheter (PICC) in 47% (31/66) CVC-VTEs, partially implanted catheter in 42% (28/66), non-implantable catheter in 7% (5/66), and totally implanted catheter (Port) in 2% (1/66). The most used CVC caliber was 2–4 Fr (31/52, 60%), followed by 5–7 Fr (12/52, 23%). In 52% of cases (32/61), the lumen was single, in 46% (28/61), it was double, and in 2% (1/61), it was a trilumen. The material was silicone in 56% (29/52) and polyurethane in 44% (23/52).

**Table 3B:** CVC characteristics.

**CVC insertion technique (data available in 66/78)**	Percutaneous: 56/66 (85%) Surgical: 10/66 (15%)
**CVC type (data available in 66/78)**	PICC: 31/66 (47%) Partially implanted: 29/66 (43%) Non-implantable: 5/66 (7%) Totally implanted (Port): 1/66 (2%)
**CVC side (data available in 78/78)**	Right: 53/78 (68%) Left: 25/78 (32%)
**CVC caliber (data available in 52/78)**	2–4 Fr: 31/52 (60%) 5–7 Fr: 12/52 (23%) 8–13.5 Fr: 9/52 (17%)
**Number of lumens (data available in 61/78)**	1: 32/61 (52%) 2: 28/61 (46%) 3: 1/61 (2%)
**CVC material (data available in 52/78)**	Silicone: 29/52 (56%) Polyurethane: 23/52 (44%)

*CVC, central venous line; VTE, venous thrombotic event; ICTE, intracardiac thrombotic event; IQR, interquartile range; PICC, peripherally inserted central catheter*.

### Thrombophilia

In 71% (54/76) of patients, no family history for thrombotic events was reported. The methods and the normal range values of the thrombophilic tests performed are detailed in Supplementary Table 1.

One or more thrombophilia tests were carried out in 60% (46/76) of patients, at the onset of CVC-VTEs.

A search for genetic thrombophilia (factor V Leiden and prothrombin G20210A mutations) was performed only in 26% (20/76) of patients, and only one had a heterozygous G20210 mutation.

Increased factor VIII was reported in 9% (4/46) of patients, hyperhomocysteine in 4% (2/46), and an elevated lipoprotein (a) level in 2% (1/46).

Antithrombin deficiency (levels lower than 72% in infants and 90% in children aged more than 1 year) were reported in 15% (7/46) of cases, protein C deficiencies in 13% (6/46) of cases, and protein S defects (one homozygote) in 9% (4/46). The lupus anticoagulant (LAC) was tested in 6 patients, and none was positive.

### Treatment

An anti-thrombotic treatment was administered in 96% of CVC-VTEs (75/78) (Table 4); in the remaining 4% (3/78), the treatment was not administered due to a delayed diagnosis in asymptomatic patients.

**Table 4:** Data on treatment of systemic CVC-VTEs (data available in 75/78).

**Therapy**	**Non-cardiac VTEs (*n* = 67)**	**ICTEs (*n* = 11)**
**UFH**	3	1
**LMWH**	47	3
**UFH** **+** **LMWH**	5	2
**rt-PA** **+** **LMWH**	2	0
**rt-PA** **+** **UFH** **+** **LMWH**	1	0
**Urokinase** **+** **LMWH**	1	0
**rt-PA** **+** **UFH**	0	1
**Urokinase** **+** **UFH**	0	1
**UFH** **+** **Warfarin**	2	0
**Warfarin**	3	0
**ASA**	3	1
**Supportive treatment**	5	1

*ASA, acetylsalicylic acid; CVC, central venous catheter; LMWH, low-molecular-weight heparin; rt-PA, recombinant tissue plasminogen activator; UFH, unfractioned heparin*.

Heparin was the most common treatment used (72/78, 91%), particularly subcutaneous low- molecular-weight heparin (LMWH) (61/78, 78%). LMWH was used as a monotherapy in 64% (50/78) and associated with other drugs in 14% (11/78). Intravenous unfractionated heparin (UFH) was used as a monotherapy in 5% (4/78) cases (3 non-cardiac VTEs in femoral and subclavian veins and 1 ICTE); 2 of these patients had surgery, 1 neoplasia, and 1 infection. In 10% (8/78) CVC-VTEs (6 non-cardiac VTEs in jugular and femoral veins and 2 ICTEs), both subcutaneous LMWH and intravenous UFH were used; 3 had neoplasia, 2 had surgeries, and the remaining had dehydration, infection, and cardiopathy.

The types of LMWHs used were enoxaparin in 57% (35/61), nadroparin in 23% (14/61), and dalteparin in 20% (12/61). The data on the length of LMWH treatment was available in 45/61 events: the overall median duration of anticoagulation was 31 days (range 7–270 days) with the following age group differences: median 31 days in infants <2 years (range 13–114 days) and 42 days in adolescents (range 6–270 days). An LMWH treatment longer than 3 months was given only in 4 oncological patients with very extensive thrombosis. Anticoagulation was discontinued due to thrombus resolution (partial or complete), evaluated by D-US.

Antithrombotic prophylaxis with UFH or LMWH was administered in 10 patients (12%) affected by the following risk factors: cardiopathy in 3, infections in 3, neoplasm in 2, and short bowel syndrome in 2.

Oral anticoagulation with warfarin alone was given in 3 patients affected by chronic renal failure, all with VTEs in the subclavian vein; warfarin was associated with UFH in 2 cases with chronic diseases (congenital cardiopathy and inflammatory bowel disease) and femoral thrombosis.

Fibrinolytic therapy was administered in 6 CVC-VTEs: systemic fibrinolysis was performed in 4 oncologic cases (3 non-cardiac VTEs and 1 ICTE), with extended venous thrombosis and life-threatening events and/or the risk of limb viability; local fibrinolytic therapy was administered in 2 cases with the thrombus adjacent to the catheter site (one with neoplasia and ICTE and the one with post-surgical sepsis and VTEs). The used agents for fibrinolysis were as follows: a low-dose recombinant tissue plasminogen activator (rt-PA) (0.03–0.06 mg/kg/h) in 2 events and urokinase with a starting bolus dose of 4,000 IU/kg in 4 events. The duration of this therapy ranged from 1 h to 6 days with a median of 48 h. Indeed, one patient with ICTE, scarcely responding, received three administrations (for 48 h at first, subsequently repeated twice) with a prolonged fibrinolytic treatment of 6 days; this case received a very low dose of rt-PA (0.03 mg/kg/h). All the 6 events treated with thrombolysis were subsequently started on heparin, and 4 had a complete resolution of thrombus.

Antiplatelet therapy with acetylsalicylic acid was administered only in 4 cardiopathic cases with CVC-VTEs (5%). Supportive treatments (fresh-frozen plasma and antithrombin therapy), as well as anticoagulation, were administered in 6 cases. Thrombectomy was performed in one patient with massive superior vena cava thrombosis. No therapy-related adverse events or bleeding was reported.

### Outcome

A second thrombotic event, radiologically documented, was reported in 2.6% (2/76) of patients (one with neoplasm and one with inflammatory bowel disease). These events occurred during antithrombotic therapy in a different site with respect to the first episode, respectively 4 and 2 weeks later. The outcome at discharge, considering radiological clot resolution and/or the signs suggesting that clinical sequelae was available in 37/78 events: in 70% (24/37), no sequelae were reported. In the remaining 30% (10/37), PTS was reported in 5 events, CVC replacement in 4, and ischemic necrosis with toe finger amputation in 1. Three patients died due to an underlying disease; no CVC-VTE-related deaths were reported.

The data on a 3-month follow-up were available in 20/78 events: total or partial clot resolution without any permanent issue was reported in 75% (15/20) events.

The data on a 12-month follow-up was available in 10/78 events only: in 3 events, there was a total or partial clot resolution, while 1 recurrence was reported. PTS was reported in 4 out of 5 patients having this complication at discharge (one patient was lost at a follow-up). None had PE. Death, unrelated to thrombosis, occurred in one.

## Discussion

This is the first report on pediatric systemic CVC-VTEs from the RITI.

The presence of CVCs represents a well-recognized major predisposing factor for thrombosis development in children (1, 4, 5, 9, 19–22). Although our registry RITI cannot be used for epidemiological purposes, Box 1 shows the proportion of CVCs among pediatric VTEs in some of the main registries and nationwide studies on pediatric VTEs (4, 12, 23, 24).

Box 1 **Data on CVC-VTEs from the main registries or nationwide studies on pediatric systemic venous thromboembolism (VTE).**

**Table:** 

	**Andrew et al. (4)**	**van Ommen et al. (12)**	**Tuckuviene et al. (23)**	**Jaffray et al. (24)**
**Country**	**Canada**	**The Netherlands**	**Denmark**	**United States**
**Number of children with systemic VTE**	137	99 (47 neonates, 52 children)	331	621
**Age of children with systemic VTE**	1 month−18 years	0–18 years	0–18 years	0–21 years
**Proportion of patients with CVC**	33% (45/137)	94% (44/47) in neonates 36% (19/52) in children	8% (24/297)	80% (497/621)

*Legend: CVC, central venous line; VTE, venous thrombotic event*.

### Risk Factors for VTEs

Besides CVCs, a number of other acquired and inherited risk factors for pediatric VTEs have been identified in the literature, with multifactorial etiology in over 90% (4, 19, 20). In our registry, 83% of CVC-VTEs developed in children with at least one or more acquired prothrombotic disorders, in agreement with the Canadian (96%), Dutch (98%), and Danish (86.6%) registries (4, 12, 23). Chronic systemic diseases, including malignancies, cardiopathy, renal failure, and malformative and neurometabolic diseases represented the most frequent underlying risk conditions in our CVC-VTE cohort.

Moreover, supportive treatments (transfusions and antibiotics), as well as total parenteral nutrition, were the most frequent reasons for CVC insertion in our registry; these may also predispose to thrombosis since they can induce endothelial cell damage with the release of pro-coagulant factors and platelet activation, leading to thrombus formation and deep vessel occlusion (9, 25). In particular, free fatty acids included in total parenteral nutrition have multiple effects upon endothelial cells that increase vascular thrombogenicity (26).

Similar to the literature (27), younger children, in particular under 2 years of age, represented the most affected group by CVC-VTEs in our registry. This may be partly due to a wider use of total parenteral nutrition in this age. Moreover, the traumatic damage induced by the catheter in small-sized vessels, with blood flow disruption, is an additional cause of thrombosis in infancy (28). An optimal diameter ratio of 1:3 has been suggested; however, this may not be achievable in infants and small children (27).

Inherited thrombophilia is a known factor associated with an increased likelihood of developing thrombosis, although our data are insufficient to demonstrate a relationship between thrombophilia and systemic CVC-VTEs, similar to the literature. Indeed, thrombophilic studies were carried out heterogeneously in our registry, often at onset of the CVC-VTE. The plasmatic levels of many anticoagulants (protein C, protein S, and antithrombin) may transiently decrease during acute thrombosis. Similarly, factor VIII and Lp(a) can be elevated in inflammatory conditions (29). Moreover, it should be remembered that the normal values for coagulation factors may change in different developmental stages and ages. In particular, plasma antithrombin levels are lower in the first months due to the characteristics of the neonatal hemostatic system. Canadian and Dutch registries do not report an increased prevalence of underlying thrombophilia in patients with CVC-VTEs (12, 30). Furthermore, the Kidcat study showed that underlying inherited prothrombotic conditions do not have any impact on the thrombotic risk in children with CVC (22), and similarly, thrombophilia did not predict recurrent catheter-related deep vein thrombosis in children in another recent work (31). Nevertheless, further and more homogeneous data on larger cohorts should be warranted (32).

### CVC Characteristics and Location

In our registry, the catheters were inserted percutaneously in 85% of cases. Among these cases, the patients included were those with severe conditions like sepsis, hypotensive attacks, and end-stage renal disease; in contrast, only a minority required a surgical insertion. The number of PICCs compared to tunneled central catheters (TCCs) was similar (47 vs. 44%). Despite the relative ease and simplicity of use of PICCs, leading to a substantial rise in their use, confirmed by our results, recent literature data demonstrate that such lines pose a substantial risk for VTEs, similar to (33) or higher than TCCs (34). PICC factors were reported to be associated with an increased risk of thrombosis, although inconsistently, they include repetitive PICC insertions in the same arm (35, 36), larger diameter and double-lumen PICCs (1, 36–39). Overall, though, literature data are insufficient to definitively link these factors to an increased risk of CVC-VTEs in children (1).

In our registry, CVCs were located in the lower venous system (particularly in the femoral vein) in about half of the cases, similar to other literature data (38). In our registry, 30% were PICU inpatients; in these cases, the emergency conditions and the greater ease of insertion probably explain the extensive use of femoral accesses and the greater number of thromboses in this site. The low cardiac output, the slow venous flow from the lower extremities, and the vein compression by the inguinal ligament may possibly contribute to the increase of the incidence of VTEs associated with femoral lines.

In our registry, the interval between CVC insertion and the diagnosis of CVC-VTEs was median 10 days. Recent studies have shown that the risk of thrombosis is highest during the first 4–5 days of catheter placement (2, 37, 40). This occurrence might be secondary to the traumatism of catheter insertion, which is a predisposing factor for vascular thrombosis, more relevant than other factors like catheter size, the number of lumens, and catheter material.

### Diagnostic Investigations

In our registry, 23% of CVC-VTEs were asymptomatic and were incidentally detected on the imaging performed for the underlying disease. The diagnosis of non-cardiac VTEs was most frequently done with D-US, a reliable, non-invasive, and readily available method, while all ICTEs were diagnosed by echocardiography. Despite the fact that venography was considered the gold standard to evaluate the upper venous system in the literature, it has largely been replaced by D-US in the recent years (1) and it was not usually performed as the first step in our cohort because of its more invasive nature and exposure to intravenous contrast and radiation (2). According to previous research comparing venography and ultrasound for the diagnosis of TEs in the upper body, D-US does not appear to be sensitive in detecting VTEs in the subclavian veins (due to the shadows caused by the clavicle, sternum, and lungs, as well as the inability to compress the subclavian veins secondary to the overlying clavicle), yet it appears to be more sensitive than venography in the jugular vein (2, 41). In our study, both lower and upper venous systemic thromboses were usually diagnosed with D-US. Venography was used only in 2 cases with extensive thrombosis of the superior vascular compartment. This diagnostic approach could explain our lower incidence of subclavian vein thromboses compared to the literature (37, 38, 40, 41). It is noteworthy that D-US, when performed to investigate comorbidities in patients with CVC, disclosed 9 asymptomatic CVC-VTEs. In our opinion, despite the fact that there is no consensus in the literature about the routine screening of asymptomatic patients with CVC-VTEs with D-US, children with CVC under 2 years of age, with a severe underlying disease or admitted in PICU, should be carefully evaluated in order to promptly detect CVC-VTEs and prevent long-term complications. The pediatric risk-assessment models for hospital-acquired VTEs have been developed and may help in clinical practice (27).

### Treatment

**Figure 1:**
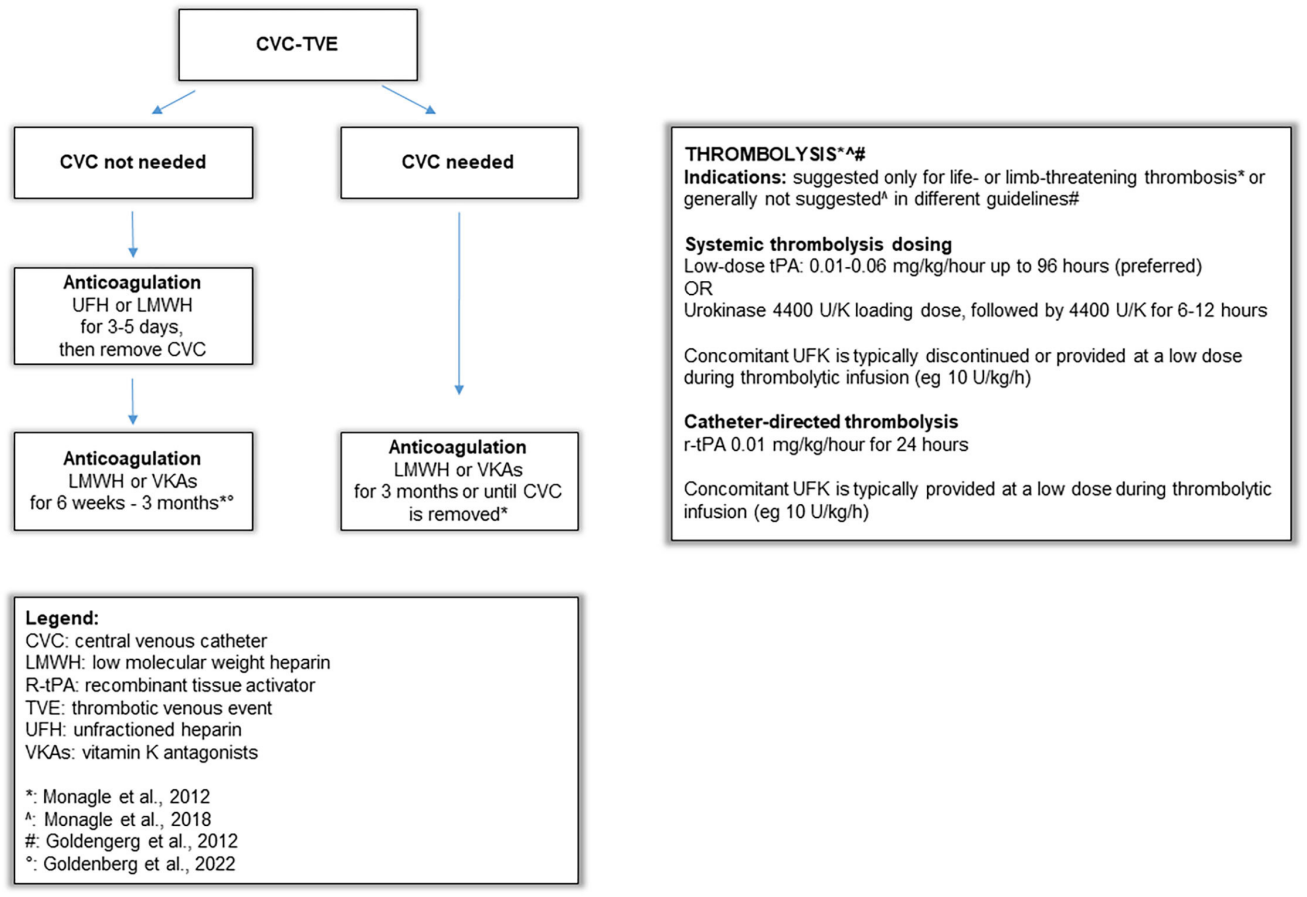
Algorithm on approach and treatment strategies of CVC-related thrombosis in children.

Supplementary Table 2 summarizes the main literature recommendations on CVC-VTE management and treatment (6, 42) and an algorithm on the approach and treatment of CVC-related thrombosis in children is shown in Figure 1. The use of antithrombotic therapy in our study was reported in 96% of our cases, with LMWH used most frequently, in agreement with previous studies in children (42, 43). The potential advantages of LMWH for children include minimal monitoring requirements and subcutaneous administration, so it is increasingly becoming an alternative to UFH (44–46). Studies on the use of direct oral anticoagulants in patients with CVC-VTE are ongoing (1). As regards the duration of anticoagulation, this was shorter in our registry (median 31 days) compared to available recommendations, where the suggested duration is ≤ 3 months (Supplementary Table 2) (6, 42); in our casuistry, the anticoagulation withdrawn was related to thrombus resolution at D-US. Interestingly, a recent randomized controlled clinical trial showed that among patients younger than 21 years of age with provoked venous thromboembolism, anticoagulant therapy for 6 weeks compared with 3 months met non-inferiority criteria based on the trade-off between a recurrent venous thromboembolism risk and bleeding risk (47).

The literature data on the efficacy and safety of routine thromboprophylaxis in preventing systemic thrombosis in children with CVC are not definitive (21, 25). Except for arterial catheters and for selected patients at increased risk of thrombosis (acute lymphoblastic leukemia or lymphoma, inherited thrombophilia, or a history of thrombotic events), the evidence-based clinical practice guidelines do not recommend the use of routine systemic thromboprophylaxis (42). Accordingly, our data showed that in 94% of Italian tertiary care pediatric centers, antithrombotic prophylaxis was not routinely used in patients with venous catheters.

### Outcome

Regarding long term-complications, it is worth noting that the PTS in our cohort was reported in a lower percentage (6.4%) than those reported in the Canadian (20%) and Danish registries (26%) of pediatric VTEs and the Goldenberg review (4, 8, 23), despite the relatively short treatment duration. In our opinion, this is a notable finding, but our short-term follow-up does not allow us to draw any definite conclusion.

## Limitations and Conclusions

The major limitation of our registry is the restricted number of patients. While the RITI is available online and open to all registered Italian physicians, not all Italian hospitals participated in the registry; therefore, RITI data cannot be used for epidemiological purposes to derive figures on the incidence and prevalence of thrombosis in Italian neonates and children. Furthermore, in the registry design, no data about CVC placement (US guided or not) have been required. Therefore, this helpful information is not available.

Moreover, since not all parts of the registry are mandatory, data availability was heterogeneous in some of the subsections.

Despite these limitations, our study is one of the few available studies focusing on CVC-VTEs in children, with data derived from a nationwide registry. Our data confirm the peak in young children for developing CVC-VTEs and provide details on the treatment of pediatric CVC-TVEs in Italy. The occurrence of asymptomatic CVC-VTEs in our registry should alert pediatricians to more carefully assess infants and severely sick children with CVCs in order to promptly detect systemic thrombosis. In particular, the pediatric risk-assessment models for hospital-acquired VTEs have been developed and may help in clinical practice (27).

RITI could represent an important tool to identify children at risk for developing VTEs and to start clinical trials targeting the prevention and better management of systemic CVC-VTEs.

## Data Availability Statement

The raw data supporting the conclusions of this article will be made available by the authors, without undue reservation.

## Ethics Statement

The studies involving human participants were reviewed and approved by Comitato Etico Padova. Written informed consent to participate in this study was provided by the participants' legal guardian/next of kin.

## The Systemic Thromboses Working Group of the Italian Registry of Pediatric Thrombosis (RITI, Registro Italiano Trombosi Infantili)

Additional members of the Systemic Thromboses Working Group of the RITI include the following:

Manuela Agostini, Bianca Bassi, Elisa Bertoni, Anna Casani, Daniela Farinasso, Elena Gallo, Chiara Gentilomo, Massimo Grassi, Fabio Lunetta, Mariella Magarotto, Francesca Maschio, Antonella Palmieri, Andrea Pettenazzo, Roberto Sangermani, Annamaria Laverda.

## Author Contributions

DL enrolled patients in the Registry, collected the data, and drafted the manuscript. MN, AM, PSa, MPe, FP, MPu, MG, PG, ST, ML, AS, AT, and DT enrolled patients in the Registry and contributed to the critical review and the final editing of the manuscript. GL, MM, AF, and DG contributed to the critical review and the final editing of the manuscript. SS and Psi had a major role in the creation of the Registry, enrolled patients in the Registry, and contributed to the critical review and the final editing of the manuscript. All authors contributed to the article and approved the submitted version.

## Conflict of Interest

The authors declare that the research was conducted in the absence of any commercial or financial relationships that could be construed as a potential conflict of interest.

## Publisher's Note

All claims expressed in this article are solely those of the authors and do not necessarily represent those of their affiliated organizations, or those of the publisher, the editors and the reviewers. Any product that may be evaluated in this article, or claim that may be made by its manufacturer, is not guaranteed or endorsed by the publisher.
