# Supplementary material for: Systemic Catheter-Related Venous Thromboembolism in Children: Data From the Italian Registry of Pediatric Thrombosis
Source: Front Pediatr. 2022 Mar 23;10:843643. doi: 10.3389/fped.2022.843643 (PMC8984174; doi:10.3389/fped.2022.843643)
Supplement: Supplementary file 1 [file Table_1.docx]

| **Supplementary Table 1** | | |
| --- | --- | --- |
| **Coagulation Inhibitors and thrombophilic factors, methods and reference values** | | |
| **Coagulation inhibitors** | **method** | **Values (range )** |
| Antithrombin: | chromogenic activity | 72-120% (infant);  90-120% (child) |
| Protein C | coagulometric activity | 80-120% |
| Protein C | chromogenic activity | 70-130% |
| Protein C antigen | ELISA | 80-120% |
| Protein S | coagulometric activity | 70-130% |
| Protein S free antigen | ELISA | 80-120% |
| Protein S total antigen | ELISA | 80-120% |
| **Thrombophilic factors** |  | |
| Anti beta2-glicoprotein I IgG |  | < 8 U/mL |
| Anti beta2-glicoprotein I IgM |  | < 8 U/mL |
| Anti cardiolipin IgG antibodies |  | < 10 U/mL |
| Anti cardiolipin IgM antibodies |  | < 10 U/mL |
