# Supplementary material for: Systemic Catheter-Related Venous Thromboembolism in Children: Data From the Italian Registry of Pediatric Thrombosis
Source: Front Pediatr. 2022 Mar 23;10:843643. doi: 10.3389/fped.2022.843643 (PMC8984174; doi:10.3389/fped.2022.843643)
Supplement: Supplementary file 2 [file Table_2.docx]

**Supplementary Table 2**

| **RECOMMENDATIONS ON CVC MANAGEMENT AND TREATMENT OF CVC-VTE IN CHILDREN** | | | |
| --- | --- | --- | --- |
|  | **CHEST 2012 Guidelines on antithrombotic treatment in pediatric age**  **[Monagle 2012]** |  | **American Society of Hematology 2018 Guidelines for management of venous thromboembolism**  **[Monagle 2018]** |
| **Type of antithrombotic treatment** | In children with first VTE (CVAD and non-CVAD related), we recommend acute anticoagulant therapy with either UFH or LMWH (Grade 1B). We recommend initial treatment with UFH or LMWH for at least 5 days (Grade 1B). For ongoing therapy, we suggest LMWH or UFH. For patients in whom clinicians will subsequently prescribe VKAs, we recommend beginning oral therapy as early as day 1 and discontinuing UFH/LMWH on day 6 or later than day 6 if the INR has not exceeded 2.0 compared with no therapy (Grade 1B). [Recommendation n° 2.22.1] | The ASH guideline panel suggests using either anticoagulation or no anticoagulation in pediatric patients with CVAD-related superficial vein thrombosis (conditional recommendation based on very low certainty in the evidence of effects). Remarks: There were very little direct or indirect data on which to base this recommendation. The collective experience of the panel suggested that, in most patients, no anticoagulation will be appropriate. However, anticoagulation seems appropriate for patients who have a CVAD line that is still functioning and who continue to need venous access, as well as in patients whose symptoms progress. [Recommendation n°16] | |
| **Duration of anticoagulation** | In children with secondary VTE (ie, VTE that has occurred in association with a clinical risk factor) in whom the risk factor has resolved, we suggest anticoagulant therapy be administered for 3 months (Grade 2C) as compared with no further therapy. In children who have ongoing, but potentially reversible risk factors, such as active nephrotic syndrome or ongoing asparaginase therapy, we suggest continuing anticoagulant therapy beyond 3 months in either therapeutic or prophylactic doses until the risk factor has resolved (Grade 2C). [Recommendation n° 2.22.3] | The ASH guideline panel suggests using anticoagulation for ≤3 months rather than anticoagulation for >3 months in pediatric patients with provoked DVT or PE (conditional recommendation based on very low certainty in the evidence of effect). Remarks: The panel noted that the exact duration for optimal anticoagulation was unknown, and there are ongoing studies comparing durations within this time frame. In cases in which the provoking factor is resolved, treatment for >3 months is unjustified. However, for patients who have persistence of the causative risk factor for provoked DVT/PE, longer anticoagulation could be considered. [Recommendation n°14] | |
| **CVC management after confirmed thrombosis** | In children with CVAD in place who have a VTE and in whom the CVAD remains necessary, we suggest after the initial 3 months of therapy, that prophylactic doses of VKAs (INR range, 1.5-1.9) or LMWH (anti-Xa level range, 0.1-0.3 units/mL) be given until the CVAD is removed (Grade 2C). If recurrent thrombosis occurs while the patient is receiving prophylactic therapy, we suggest continuing therapeutic doses until the CVAD is removed and for a minimum of 3 months following the VTE (Grade 2C). [Recommendation n° 2.22.7]  In children with a CVAD in place who have a VTE, if a CVAD is no longer required, or is nonfunctioning, we recommend it be removed (Grade 1B). We suggest at least 3 to 5 days of anticoagulation therapy prior to its removal rather than no anticoagulation prior to removal (Grade 2C). If CVAD access is required, and the CVAD is still functioning, we suggest that the CVAD remain in situ and the patient be given anticoagulants (Grade 2C). For children with a first CVAD-related VTE, we suggest initial management as for secondary VTE as previously described. [Recommendation n° 2.22.6] | The ASH guideline panel suggests no removal, rather than removal, of a functioning CVAD in pediatric patients with symptomatic CVAD-related thrombosis who continue to require venous access (conditional recommendation based on very low certainty in the evidence of effects). Remarks: The panel placed a high value on avoiding the insertion of another CVAD in children who may have limited availability of access sites and considered the thrombogenic effect of placing another line and new endothelial injury. The panel considered that treatment of symptomatic CVAD-related thrombus with anticoagulation likely leads to minimal complications. [Recommendation n°9]  The ASH guideline panel recommends removal, rather than no removal, of a nonfunctioning or unneeded CVAD in pediatric patients with symptomatic CVAD-related thrombosis (strong recommendation based on very low certainty in the evidence of effects). Remarks: In situations in which ongoing care of the primary condition can be delivered adequately without central venous access, removal of the stimulus to the thrombosis is appropriate. An overriding principle is that any central access device should be removed as soon as feasible within the confines of the overall treatment of the child. The panel made a strong recommendation despite very low certainty of evidence for benefits based on high evidence of harm or high cost. [Recommendation n°10]  The ASH guideline panel suggests delayed removal of a CVAD until after initiation of anticoagulation (days), rather than immediate removal in pediatric patients with symptomatic central venous line–related thrombosis who no longer require venous access or in whom the CVAD is nonfunctioning (conditional recommendation based on very low certainty in the evidence of effects). Remarks: The panel placed high value on avoiding potential risk of emboli leading to PE or paradoxical stroke, and this was thought to be achieved by a few days of anticoagulation. The risk of infection and bleeding with anticoagulation before removing the CVAD was considered to be small. The panel recognized that surgical availability was often a pragmatic determinant of timing of CVAD removal. [Recommendation n°11]  The ASH guideline panel suggests either removal or no removal of a functioning CVAD in pediatric patients who have symptomatic CVAD-related thrombosis with worsening signs or symptoms, despite anticoagulation and who continue to require venous access (conditional recommendation based on very low certainty in the evidence of effects). Remarks: The panel considered the variability in value placed by families and clinicians on maintaining line access compared with potential risk of infection and further thrombus progression, which will vary for individual patients. If alternative venous access is readily available, then removal of CVAD in the setting of worsening VTE symptoms, despite anticoagulation, is appropriate. However, in some children, venous access is paramount. [Recommendation n°12] | |
| **Thrombolysis** | In children with VTE, we suggest that thrombolysis therapy be used only for life- or limb-threatening thrombosis (Grade 2C). If thrombolysis is used in the presence of physiologically low levels or pathologic deficiencies of plasminogen, we suggest supplementation with plasminogen(Grade 2C). In children with VTE in whom thrombolysis is used, we suggest systemic thrombolysis or catheter-directed thrombolysis, depending on institutional experience and, in the latter case, technical feasibility. [Recommendation n° 2.23] | The ASH guideline panel suggests against using thrombolysis followed by anticoagulation; rather, anticoagulation alone should be used in pediatric patients with DVT (conditional recommendation based on very low certainty in the evidence of effects). Remarks: The panel considered issues, such as the size and clinical impact of VTE, as important in deciding the relative risk benefit ratio of thrombolysis. In most cases, the risks seem too high for the potential benefit; however, there may be individuals in whom the opposite is true. Extrapolation of adult data was difficult. There are insufficient data to address the relative risk benefit of local thrombolysis via interventional radiology compared with systemic thrombolysis, and the panel noted that the centers with access to pediatric interventional radiology were often stronger advocates of thrombolysis. [Recommendation n° 3] | |
| **Loss of CVC patency** | For CVADs, we suggest flushing with normal saline or heparin or intermittent recombinant urokinase (rUK) to maintain patency as compared with no therapy (Grade 2C). For blocked CVADs, we suggest tPA or rUK to restore patency (Grade 2C). If after at least 30 min following local thrombolytic instillation CVAD patency is not restored, we suggest a second dose be administered. If the CVAD remains blocked following two doses of local thrombolytic agent, we suggest radiologic imaging to rule out a CVAD-related thrombosis (Grade 2C). [Recommendation n° 2.30] | - | |
| **Thrombophylaxis** | For children with short- or medium-term CVADs, we recommend against the use of routine systemic thromboprophylaxis (Grade 1B). [Recommendation n° 2.31]  For children receiving long-term home TPN, we suggest thromboprophylaxis with VKAs (Grade 2C). [Recommendation n° 2.34] | - | |

Supplementary Table 2. Recommendations on CVC management and treatment of CVC-VTE in children, derived from CHEST 2012 Guidelines on antithrombotic treatment in children [Monagle 2012], and from the American Society of Hematology 2018 Guidelines for management of venous thromboembolism [Monagle 2018].

Legend: CVAD: central venous access device; CVC: central venous line; LMWH; low molecular weight heparin; rUK: recombinant urokinase; TNP: total parenteral nutrition; UFH: unfractioned heparin; VKAs: vitamin K antagonists; VTE: venous thrombotic event.
